# Strategy Shift Toward Lower Spatial Frequencies in the Recognition of Dynamic Facial Expressions of Basic Emotions: When It Moves It Is Different

**DOI:** 10.3389/fpsyg.2019.01563

**Published:** 2019-07-17

**Authors:** Marie-Pier Plouffe-Demers, Daniel Fiset, Camille Saumure, Justin Duncan, Caroline Blais

**Affiliations:** ^1^Département de Psychologie, Universtité du Québec en Outaouais, Gatineau, QC, Canada; ^2^Département de Psychologie, Université du Québec à Montréal, Montreal, QC, Canada

**Keywords:** facial expressions, basic emotion, perceptual strategy, spatial frequency tuning, dynamic advantage

## Abstract

Facial expressions of emotion play a key role in social interactions. While in everyday life, their dynamic and transient nature calls for a fast processing of the visual information they contain, a majority of studies investigating the visual processes underlying their recognition have focused on their static display. The present study aimed to gain a better understanding of these processes while using more ecological dynamic facial expressions. In two experiments, we directly compared the spatial frequency (SF) tuning during the recognition of static and dynamic facial expressions. Experiment 1 revealed a shift toward lower SFs for dynamic expressions in comparison to static ones. Experiment 2 was designed to verify if changes in SF tuning curves were specific to the presence of emotional information in motion by comparing the SF tuning profiles for static, dynamic, and shuffled dynamic expressions. Results showed a similar shift toward lower SFs for shuffled expressions, suggesting that the difference found between dynamic and static expressions might not be linked to informative motion *per se* but to the presence of motion regardless its nature.

## Introduction

In social settings, the human face represents one of the richest nonverbal sources of information. It is thus an essential skill for humans to continually monitor the facial expressions of others in order to appropriately tailor their behavior throughout social interactions. The ability to accurately extract emotional information plays a major role in prosociality (Marsh et al., 2007), and this capacity is often found to be altered in numerous psychiatric conditions characterized by impaired social functioning, such as schizophrenia (Mandal et al., 1998; Edwards et al., 2002; Lee et al., 2010; Clark et al., 2013; Kring and Elis, 2013) and autism spectrum disorder (Baron-Cohen and Wheelwright, 2004; Harms et al., 2010).

Until recently, a majority of studies investigating the visual processes underlying facial emotion recognition have relied on static pictures displaying facial emotions at their apex (i.e., highest intensity). However, facial emotions are dynamic and transient by nature; thus, the visual information necessary to recognize a facial expression in everyday life must be extracted quickly. The present study was aimed at gaining a better understanding of this process by investigating the mechanisms subtending this important endeavor, using more ecological dynamic facial expressions. More specifically, we were interested in utilization of spatial frequencies (SF), considered the “atom” upon which primary visual cortex neurons base their world representation (DeValois and DeValois, 1990), during recognition of static and dynamic facial expressions. Simply put, lower SFs code coarser visual information, such as global face shape or facial feature location, while higher SFs code finer visual information, such as facial feature shape or details like wrinkles.

Behavioral, neuroimaging, and lesion data suggest that static and dynamic facial expressions rely on partially nonoverlapping perceptual mechanisms. For instance, dynamic expressions are associated with enhanced onlooker facial muscular reactions (Weyers et al., 2006; Rymarczyk et al., 2011), and they are also better recognized than static expressions (Wehrle et al., 2000; Kamachi et al., 2001; Ambadar et al., 2005; Bould and Morris, 2008; Hammal et al., 2009; Cunningham and Wallraven, 2009a; Chiller-Glaus et al., 2011; Recio et al., 2011; see however Kätsyri and Sams, 2008; Fiorentini and Viviani, 2011; Gold et al., 2013; Widen and Russell, 2015). In addition, neuroimaging studies have shown that dynamic expressions, compared to static ones, lead to a greater activation of many structures involved in facial emotion processing (Kilts et al., 2003; LaBar et al., 2003; Sato and Yoshikawa, 2004; Schultz and Pilz, 2009; Trautmann et al., 2009; Recio et al., 2011). Crucially, dynamic expressions engage areas of the magnocellular-dorsal pathway to a greater extent than static ones (e.g., area MT; Schultz and Pilz, 2009). This parallels the findings from studies performed on patients with ventral visual stream lesions, whom exhibit dramatically impaired recognition of static emotions (Adolphs et al., 1994; Humphreys et al., 2007; Fiset et al., 2017), but a relatively preserved ability to recognize dynamic emotions (Humphreys et al., 1993; Adolphs et al., 2003; Richoz et al., 2015).

Interestingly, the magnocellular-dorsal pathway is associated with processing of motion and shows a higher sensitivity to lower SFs (Livingstone and Hubel, 1988), which might explain these various findings pertaining to dynamic emotion recognition. In contrast, the parvocellular-ventral pathway, which encompasses most of the areas involved in static face processing, is associated with processing of typically higher SF information (Livingstone and Hubel, 1988). Seeing as static and dynamic emotion recognition may rely on partially nonoverlapping cortical structures, one might expect this to be reflected in different visual information extraction strategies, namely a reliance on lower SFs during the processing of dynamic expressions compared to static ones.

Previous work by our team and others also feeds this hypothesis according to which dynamic facial emotion recognition might rely on comparatively lower SFs – though this prediction has not been explicitly tested. Indeed, although diagnostic (i.e., relevant) facial features are mostly the same for static and dynamic expressions (namely, the eyes and mouth), eye fixation patterns underlying the extraction of these features differ. Specifically, participants spend more time directly fixating diagnostic features for static expressions, whereas they spend more time fixating the center of the face (i.e., nose) for dynamic expressions (Buchan et al., 2007; Blais et al., 2012, 2017; see however, for videos of longer duration, Calvo et al., 2018). Seeing as diagnostic features will be processed in parafoveal vision for dynamic expressions viewed at a conversational distance (i.e., face span of approx. 6–14°; Yang et al., 2014) and that sensitivity to high SFs monotonically decreases with foveal eccentricity (Hilz and Cavonius, 1974), viewing dynamic (vs. static) expressions is likely to induce a shift away from higher SFs and toward lower SFs.

The finding of different patterns of eye fixations for static and dynamic expressions also begs the question of what the underlying cause might be for such an outcome. One possibility is that dynamic expressions convey additional information through motion, thereby reducing the need to extract precise feature representations coded in higher SFs – which requires foveal processing, and thus, direct fixation. A role for motion has been supported by computational studies showing that information it conveys drastically increases performance of artificial vision systems (e.g., Jiang et al., 2011, 2014). The fact that human performance during dynamic facial emotion recognition is resistant to spatial information degradation (e.g., texture and shape) as long as motion contained within expressions is preserved (e.g., exhibited by point-light displays; Cunningham and Wallraven, 2009b), and that performance is reduced when the emotion unfolding sequence (i.e., video frame order) is shuffled or reversed (Cunningham and Wallraven, 2009a), is also a strong argument in favor of motion conveying crucial information for emotion recognition.

Although many studies have supported the importance of motion for expression processing, it is possible that the different patterns of eye fixations observed for static and dynamic expressions are not necessarily for the purpose of using emotion information that is conveyed by motion. Another possibility is instead that the mere presence of motion could activate mechanisms aimed at processing it, regardless of the emotion information it may or may not convey. Such mechanisms may involve changes in eye fixation patterns, since retinal periphery is more efficient at processing temporal variations and motion (Takeuchi et al., 2004; Thompson et al., 2007; Gurnsey et al., 2008).

In other words, fixating dynamic emotional faces in their center may serve the purpose of optimizing the processing of emotion information conveyed through motion by projecting this content in parafoveal regions of the retina. Or, the change in eye fixation pattern may instead be reflexive and caused by the mere presence of motion – irrespective of the information it might convey. In turn, the SF shift hypothesized above could very well be a consequence of fixation optimization for motion processing.

The objective of the present study was twofold. First, we wished to verify the hypothesis according to which the recognition of dynamic and static facial expressions relies on partially nonoverlapping SFs by comparing tuning profiles for both types of expressions (Experiments 1, 2). Second, we wanted to verify if changes in SF tuning curves are specific to the presence of informative motion by comparing the SF tuning profiles for static, dynamic, and shuffled dynamic expressions (Experiment 2).

## Experiment 1

The SF Bubbles method (Willenbockel et al., 2010a, 2012, 2013; Thurman and Grossman, 2011; Tadros et al., 2013; Royer et al., 2017) was used in order to compare SF utilization in two different facial emotion recognition conditions: static and dynamic expressions. Although filtering faces may create stimuli that differ from what observers consciously perceive in everyday life, it directly manipulates the visual information considered as the atom of visual perception according to the dominant theory in the field of vision (DeValois and DeValois, 1990).

The SF Bubbles method consists in creating, trial-by-trial, random SF filters that are applied to an image – here, one depicting a facial expression. Participant accuracy with each filtered image is then used to infer which SF increases the likelihood of a correct answer (see *Stimuli* section for more details). This method presents important advantages in comparison with the fixed low-pass and high-pass filters that are frequently used to tackle the SF processing during facial emotion recognition (e.g., Vuilleumier et al., 2003). First, instead of simply comparing performance with low vs. high SFs, it allows to measure the complete SF tuning curve of participants. This is particularly important for tasks involving face processing, since it has been shown that sensitivity peaks at SFs between 8 and 16 cycles per face (Näsänen, 1999; Gaspar et al., 2008). Removing those frequencies from the stimuli, as is often done with low-pass and high-pass filter, may thus tap into visual mechanisms that are not specialized for face processing. Relatedly to this last point, a second important advantage of the SF Bubbles method is that, contrary to fixed filters, it does not require an (often arbitrary) decision on where the cutoffs should be applied for the low-pass and high-pass filters; in other words, what SFs should be included in the low-pass (or high-pass filters). Such decision may have a huge impact on the results. SF Bubbles make no *a priori* decision regarding such cutoffs; it simply randomly samples all of the SFs contained in a stimulus and measure performance with all of these random filters.

### Materials and Methods

#### Participants

Twenty participants (4 males; 22.8 years old on average; SD = 3.24) took part in Experiment 1. The number of participants was chosen based on previous experiments using similar methods (Willenbockel et al., 2010a; Royer et al., 2017; Tardif et al., 2017). Because the method relies on random sampling of visual information, a high number of trials are required to obtain a reasonable signal-to-noise ratio. Studies using SF Bubbles have typically relied on a high total number of trials (i.e., across participants) ranging between 10,800 (Tadros et al., 2013) and 34,500 trials (Estéphan et al., 2018) per condition (see also Tardif et al., 2017, 33,000 trials and Royer et al., 2017, 19,200 trials). The present experiment contained a total of 39,200 trials per condition thus having enough trials to obtain very stable SF tuning for each condition. All participants had normal or corrected-to-normal visual acuity and were naïve to the purpose of the experiment.

#### Stimuli

The stimuli consisted of videos and photos of 10 actors (5 males) expressing the six basic emotions (i.e., anger, disgust, fear, joy, sadness, surprise; Ekman and Friesen, 1975) as well as neutrality. Stimuli were taken from the STOÏC database (Roy et al., 2007). Videos had a duration of 450 ms and were composed of 15 frames with a duration of 30 ms each. They started with a neutral facial expression and ended at the apex of the expression. Photo stimuli were generated by extracting the last frame from the videos (i.e., the apex). Static and dynamic stimuli were spatially aligned on the main internal features (eyes, nose, mouth) across facial expressions and across actors using linear manipulations such as translation, rotation, and scaling. Additionally, dynamic stimuli were temporally aligned. Faces were cropped to exclude non-facial cues, and they were equated on mean luminance using the SHINE toolbox (Willenbockel et al., 2010b).

**Figure 1:**
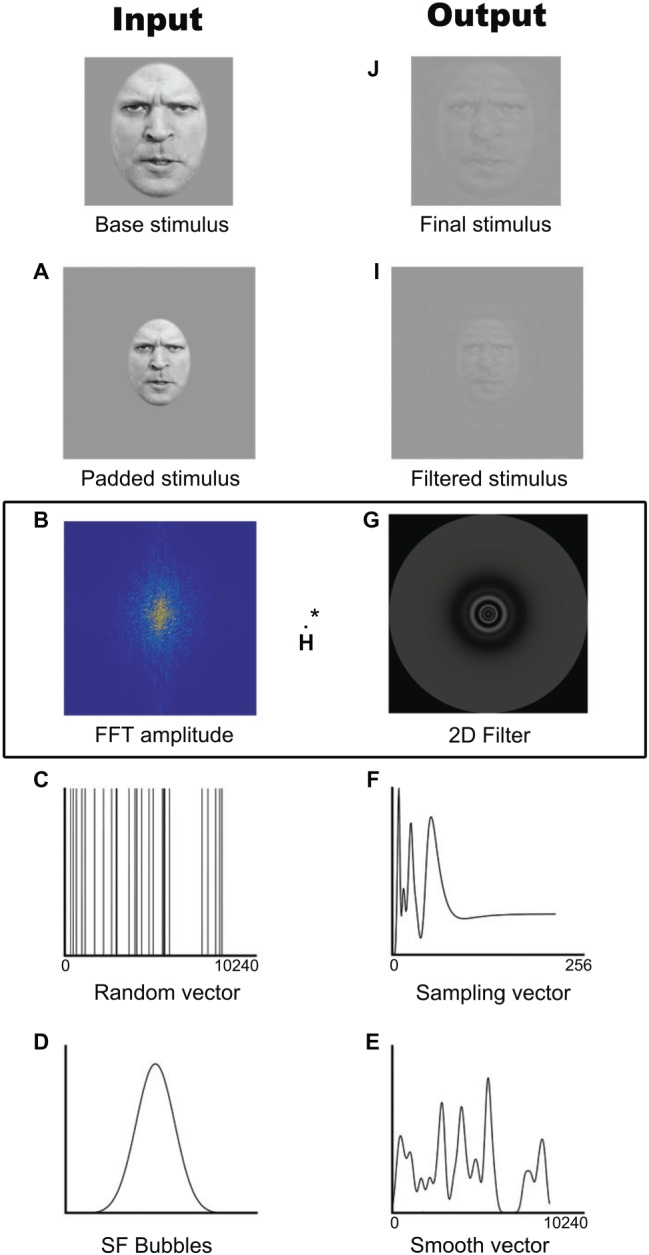
Example of the creation of one stimulus with the SF Bubbles method. **(A)** Padded stimulus. **(B)** Fast Fourrier transformed base image amplitude spectrum. **(C)** Random binary vector. **(D)** Spatial frequency Bubble. **(E)** Smoothed sampling vector. **(F)** Log-transformed sampling vector. **(G)** Two-dimensional isotropic spatial frequency filter. **(H)** Pointwise multiplication of the Fast Fourrier transformed base image amplitude spectrum and the spatial frequency filter. **(I)** Filtered stimulus. **(J)** Final cropped stimulus. Written informed consent was obtained from the individual for the publication of this image.

**Figure 2:**
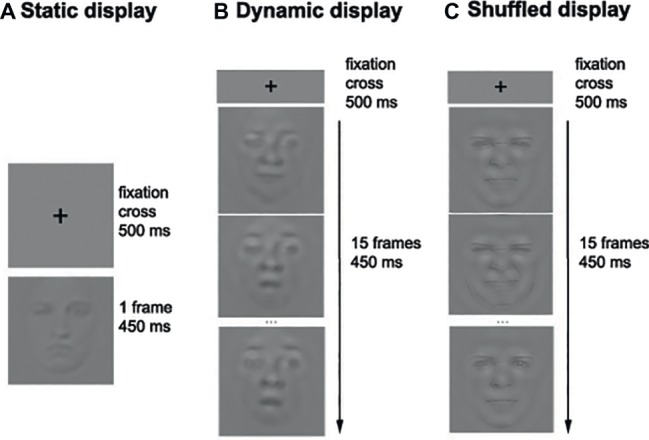
Example of the sequence of events in the Static **(A)**, Dynamic **(B)**, and Shuffled **(C)** conditions. Note that only three frames out of 15 are represented in the Dynamic and Shuffled conditions.

On each trial, a stimulus was generated by randomly sampling the SFs of the photo or the frames of the video using the SF Bubbles technique (Willenbockel et al., 2010a). This technique involves the following steps, also depicted in Figure 1. First and foremost, in order to reduce edge artifacts, the stimulus is padded with a uniform gray background (Figure 1A). A fast Fourier transform is then applied to the padded stimulus (Figure 1B), resulting in the base image amplitude spectrum to which a random SF filter is later applied. This filter is created by first generating a random binary vector of X ones among 10,240 zeros, where X is the number of bubbles (Figure 1C). This vector is then convolved with a Gaussian kernel with a standard deviation of1.5 cycles per image (Figure 1D). The smoothed sampling vector (Figure 1E) is then log-transformed in order to fit the human contrast sensitivity function (Figure 1F; see DeValois and DeValois, 1990). The resulting vector is used to generate a two-dimensional isotropic SF filter (Figure 1G) by rotating it 360° on its origin. A pointwise multiplication is performed between the base image amplitude spectrum and the SF filter (Figure 1H). The result is then back-transformed into the image domain by submitting it to an inverse fast Fourier transform (Figure 1I) and cropped to its original size (Figure 1J). The resulting “SF bubblized” image contains a random subset of the base image’s SF content. Note that with videos, the same filter was applied to all the frames within a trial. Examples of stimuli are presented in Figure 2.

#### Apparatus

The faces in all pictures and videos were presented within a square subtending 256 × 256 pixels and were displayed on a calibrated LCD monitor (51 × 28.5cm; resolution of 1,920 × 1,080) with a refresh rate of 100Hz. All participants were asked to place their head on a chin rest at a viewing distance of 38 cm; face width (about 176 pixels) subtended ≈7° of visual angle. The experimental program was written in Matlab (MathWorks, 2012), using functions from the Psychophysics Toolbox (Brainard and Vision, 1997; Pelli, 1997; Kleiner et al., 2007).

#### Procedure

Each participant completed 14 blocks of 140 trials per condition (i.e., Static and Dynamic), for a total of 3,920 trials. The experiment took on average 4 h per participant that was divided into two sessions taking place on separate days. During each session, the participants were encouraged to take breaks whenever they felt some fatigue. On each trial, a fixation cross was first displayed in the middle of the screen for 500 ms, followed by the stimulus (picture or video) for a duration of 450 ms. A uniform gray background was then displayed until the participant’s response. Participants were asked to categorize the emotion displayed by static or dynamic facial expressions by pressing the button associated with each of the six basic emotions as well as neutrality (e.g., “A” for anger, “D” for disgust, “F” for fear, etc.). Figure 2 shows the sequence of events within one trial.

All participants started with a block containing dynamic expressions and alternated between conditions thereafter. This order was kept for all participants for a specific reason. When using SF Bubbles method, the number of bubbles is manipulated with the objective of maintaining the performance between ceiling and floor. In fact, the analysis procedure allows to infer the SF utilization by comparing the SFs that were available in the stimuli on correct and incorrect trials – hence, it is imperative that a significant number of mistakes is made. In the present experiment, we decided to use the same number of bubbles with dynamic and static expressions in order to ensure that any difference found in SF tuning could not be attributable to a between-condition difference in the number of sampled SFs on each trial. We also decided to adjust the number of bubbles based on the average accuracy with dynamic expressions to minimize the likelihood of a ceiling effect, as previous studies have revealed better performance with these vs. static ones. Thus, for each participant, the number of bubbles was adjusted on a trial basis with QUEST (Watson and Pelli, 1983), but only during the blocks that contained dynamic expressions. The target average accuracy was set to 70%. The number of bubbles used on a given Static block was set to the last output of QUEST in the immediately preceding Dynamic block.

The protocol of this experiment was approved by the Research Ethics Committee of Université du Québec en Outaouais and was conducted in accordance with the Code of Ethics of the World Medical Association (Declaration of Helsinki). All participants provided informed written consent.

### Results

#### Accuracy

An average of 14.4 (SD = 13.5) bubbles was necessary to maintain an approximate accuracy of 70% during the recognition of dynamic expressions. The number of bubbles reflects the quantity of SF information (and, as a result, the total amount of energy contained in the stimulus) needed by the participants.

**Figure 3:**
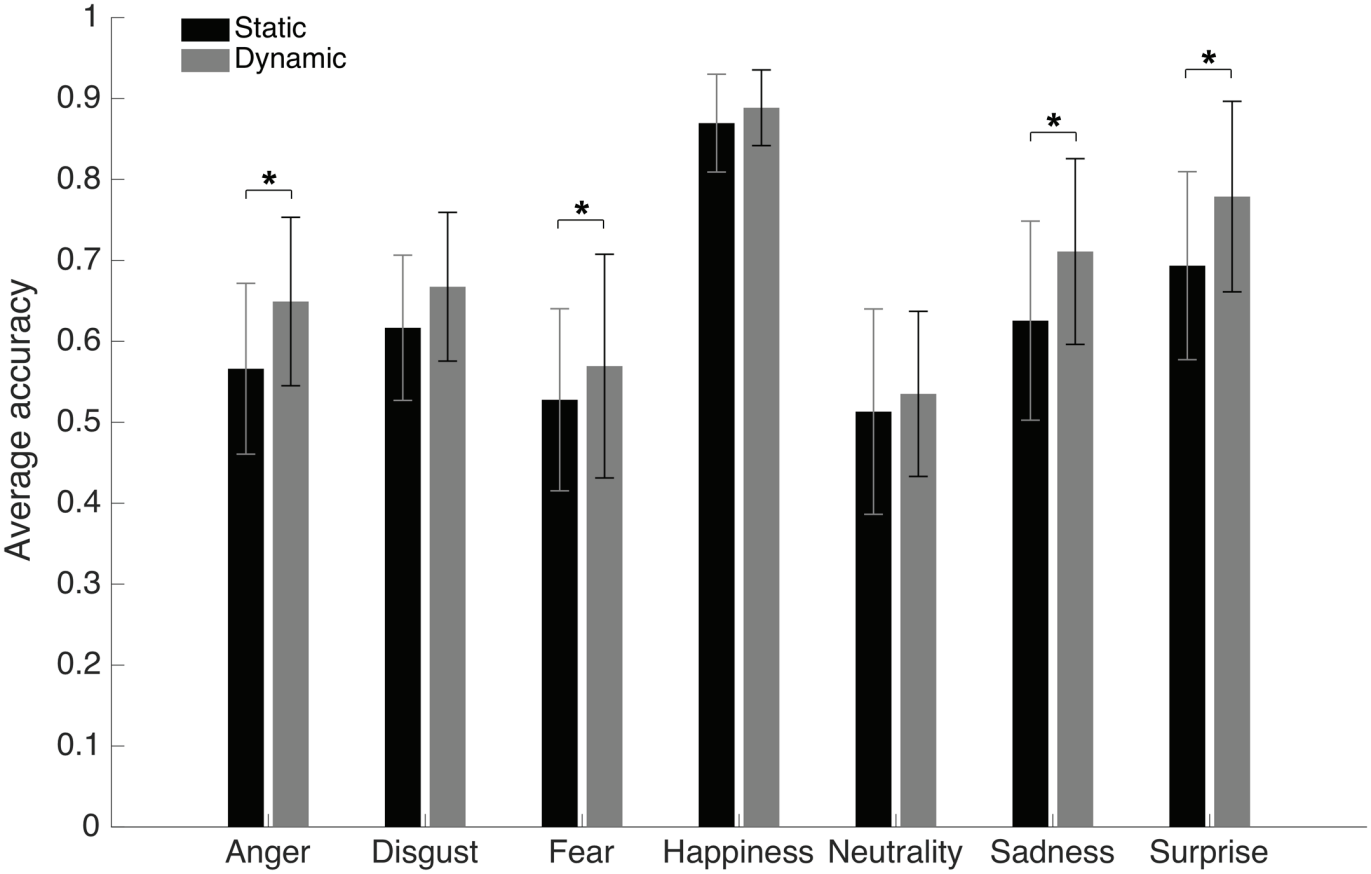
Average accuracy for the static and dynamic facial expressions in Experiment 1. Error bars represent the standard deviations. ^*^Significant at a *p* <0.007.

An average accuracy of 62.6% (SD = 4.8%) and 68.1% (SD = 5.5%) was found in the Static and Dynamic conditions, respectively. The average accuracy with each emotion in each condition is displayed in Figure 3. A 7 (Emotions) × 2 (Conditions) repeated-measure ANOVA was conducted on accuracy. The results indicated significant main effects of the factors of Emotion [*F*(1, 19) = 72.6, *p* < 0.001; *η*^2^ = 0.79] and Condition [*F*(6, 114) = 30.8, *p* < 0.001; *η*^2^ = 0.62]. There was also an interaction effect between both factors [*F*(6, 114) = 5.63, *p* < 0.001; *η*^2^ = 0.23]. A dynamic advantage was found for most facial expressions: anger [*t*(19) −8.7049; *p* < 0.001; 95% CI (−10.30 to −6.31%)], fear [*t*(19) −3.3401; *p* = 0.0034; 95% CI (−6.77 to −1.55%)], sadness [t(19) −5.2577; *p* < 0.001; 95% CI (−11.94 to −5.14%)], and surprise [*t*(19) −7.4219; *p* < 0.001; 95% CI (−10.94 to −6.13%)]. The effect for disgust did not resist the Bonferroni adjustment (*p* must be <0.007) [*t*(19) −2.6413; *p* = 0.0161; 95% CI (−9.09 to −1.05%)]. No significant effect was found for happiness [*t*(19) −2.0472; *p* = 0.0547; 95% CI (−3.84 to 0.04%)]. There was also no significant difference with neutrality [*t*(19) −1.8383; *p* = 0.0817; 95% CI (−4.69 to 0.30%)], which is normal considering the absence of motion even in the dynamic stimuli.

#### Spatial Frequency Tuning

SF tunings for static and dynamic expressions were obtained separately for each participant by calculating a weighted sum of all the unsmoothed SF vectors that were used during testing (see Figure 1C), using accuracies transformed into z-scores as weights (see Willenbockel et al., 2010a; Royer et al., 2017; Tardif et al., 2017; for a similar procedure). Thus, positive weights were granted to SF vectors that led to correct responses and negative weights were given to SF vectors that led to incorrect responses. The resulting classification vectors were smoothed using a Gaussian kernel with a standard deviation of 2.5 cycles per image and then log-transformed. Finally, they were transformed into z scores using a permutation procedure whereby weights were randomly redistributed across trials and random classification vectors were created using these weights. This procedure was repeated 20 times, and the average and standard deviation for each SF across these random classification vectors were used to standardize the coefficients obtained for each SF in the participant’s classification vector.

Group classification vectors were then produced for each condition by summing individual vectors across participants and dividing the outcome by the square root of the number of observers. The statistical threshold was determined with the Pixel test from the Stat4Ci toolbox (Zcrit = 3.1, *p* < 0.025; Chauvin et al., 2005). This threshold corrects for the multiple comparisons across SFs, while also taking into account the non-independence between contiguous SFs.

**Figure 4:**
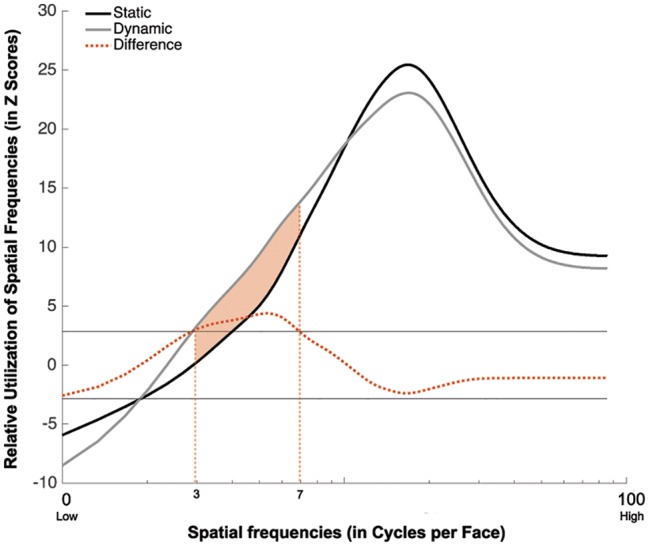
Association between the availability of a given SF and participants accuracy for recognizing static (in black) and dynamic (in gray) expressions. This association is averaged across all participants and emotions. The dotted red line represents the difference between the Dynamic and Static conditions. The SFs that are significantly more used in the Dynamic than Static condition are indicated by the shaded orange area between the curves.

Group classification vectors are displayed in Figure 4. A SF tuning peaking at 18.0 cycles per face (cpf) with a full width at half maximum (FWHM) of 30.3 cpf was found in the Static condition, and a SF tuning peaking at 17.3 cpf with a FWHM of 29.3 cpf was found in Dynamic condition. Most importantly, a significant difference in tuning was found between 3 and 7 cpf, indicating that this information was used more efficiently in the Dynamic vs. Static condition.

### Discussion

The results of Experiment 1 show a shift toward lower SFs for dynamic compared to static expressions. This shift was expected based on the differences previously observed in the eye fixation pattern used with dynamic and static expressions. Experiment 2 aimed at verifying if the difference observed in the SF tuning is related to the presence of informative motion in dynamic expressions.

## Experiment 2

### Materials and Methods

#### Participants

Twenty-eight participants (9 males; 23 years old on average; SD = 5.77), none of whom participated in Experiment 1, were tested in Gatineau (Quebec, Canada). The number of participants was selected in order to match the total number of trials per condition in Experiment 1. However, to avoid an excessive increase in the duration of the experiment due to the addition of a third condition, we decreased the number of trials that a participant needed to complete in each condition and increased the number of participants. All participants had normal or corrected-to-normal visual acuity.

#### Stimuli

The same stimuli as in Experiment 1 were used in the Static and Dynamic conditions. In the Shuffled condition, the stimuli were created by randomizing the order of the 15 frames contained in the original dynamic stimuli.

#### Apparatus

Same as in Experiment 1.

#### Procedure

Each participant completed 10 blocks of 140 trials in each condition, for a total of 4,200 trials. The unfolding of events in a trial was the same as in Experiment 1 (see Figure 2). The participant’s task was also the same as in Experiment 1.

All participants started with a block from the Dynamic condition, followed by a block from the Static condition and by a block from the Shuffled condition. The three conditions were then interleaved, and the same order was kept for the rest of the experiment. As was done in Experiment 1, the number of bubbles was adjusted on a trial basis, using QUEST during the Dynamic condition; the same number of bubbles was then applied for the following Static and Shuffled blocks.

The protocol of this experiment was approved by the Research Ethics Committee of Université du Québec en Outaouais and was conducted in accordance with the Code of Ethics of the World Medical Association (Declaration of Helsinki). All participants provided informed written consent.

### Results

#### Accuracy

**Figure 5:**
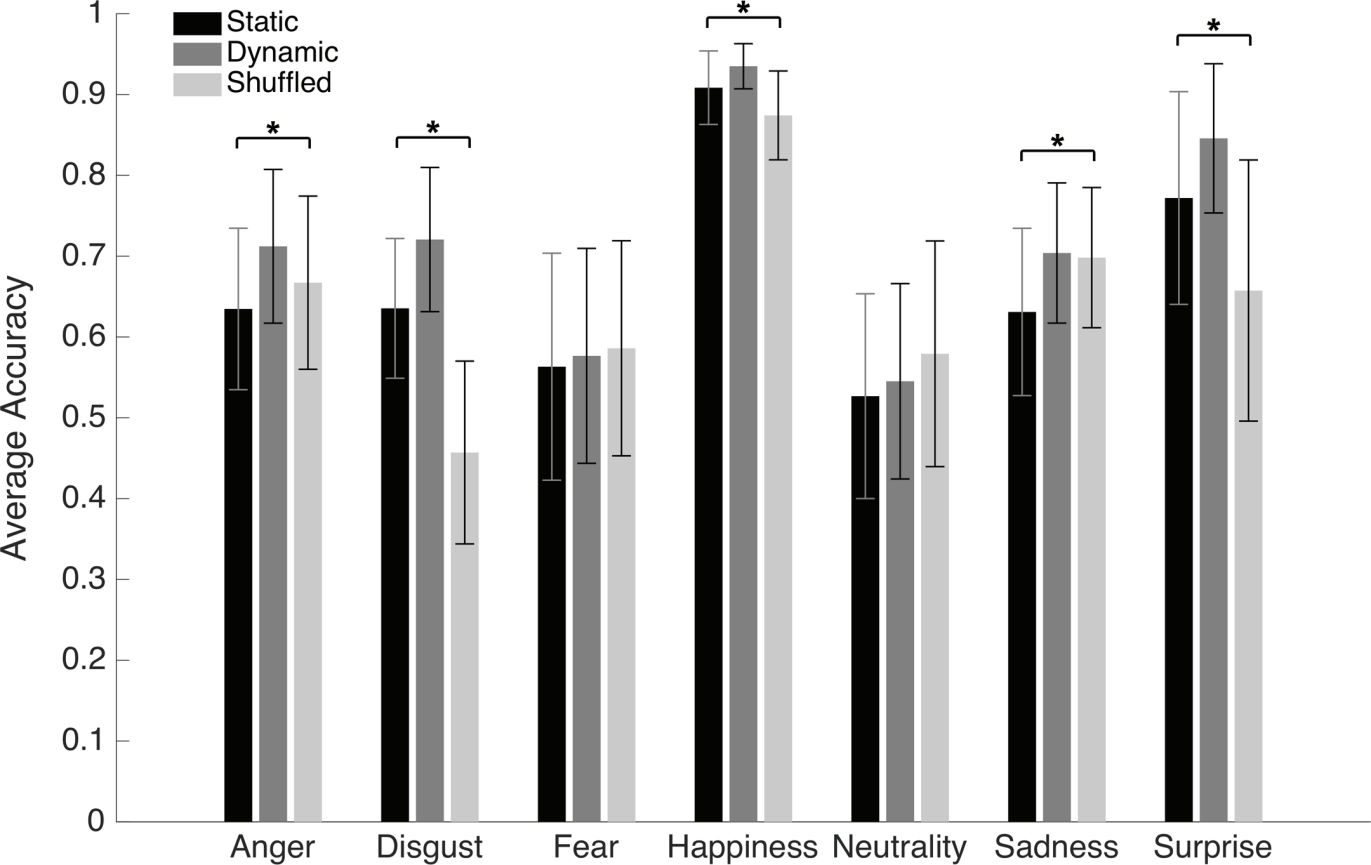
Average accuracy for the static, dynamic, and shuffled facial expressions in Experiment 2. The error bars represent the standard deviations. ^*^Significant at a *p* <0.007.

An average of 13.6 (SD = 4.15) bubbles was necessary to maintain an approximate accuracy rate of 70% in the Dynamic condition. An average accuracy of 66.5% (SD = 2.4%), 71.7% (SD = 2.2%), and 64.3% (SD = 2.7%) was found in the Static, Dynamic, and Shuffled conditions, respectively. The average accuracy with each emotion in each condition is presented in Figure 5. A 7 (Emotions) × 3 (Conditions) repeated-measure ANOVA was conducted on accuracy. The results indicated significant main effects of the factors of Emotion [*F*(6, 162) = 37.4, *p* < 0.001; *η*^2^ = 0.58] and Condition [*F*(2, 64) = 201.2, *p* < 0.001; *η*^2^ = 0.88]. These were characterized by the presence of an interaction effect between both factors [*F*(12, 324) = 37.1, *p* < 0.001; *η*^2^ = 0.58]. One-way ANOVAs were then performed for each emotion. A significant effect of condition was found for disgust [*F*(2) = 53.8, *p* < 0.001; *η*^2^ = 0.57], happiness [*F*(2) = 13.3, *p* < 0.001; *η*^2^ = 0.25], sadness [*F*(2) = 5.3, *p* = 0.007; *η*^2^ = 0.12], and surprise [*F*(2) = 14.6, *p* < 0.001; *η*^2^ = 0.27]. With anger, the effect of Condition did not resist the Bonferroni adjustment (*p* must be <0.007) [*F*(2) = 4.2, *p* = 0.019; *η*^2^ = 0.09]. No significant effect of condition was found for fear [*F*(2) = 0.20, *p* = 0.82] or neutrality [*F*(2) = 1.2, *p* = 0.31]. For the four emotions showing a significant effect of Condition, as well as for anger (for which there was an effect prior to the Bonferroni adjustment), paired sample *t*-tests were carried to contrast accuracy for Dynamic vs. Static, Dynamic vs. Shuffled, and Static vs. Shuffled. The detailed results are provided in Table 1. Overall, participants were significantly more accurate in the Dynamic (vs. Static) condition for the emotions of anger, disgust, happiness, surprise, and sadness. They were also significantly more accurate in the Dynamic (vs. Shuffled) condition for the emotions of anger, disgust, happiness, and surprise, but not sadness. Finally, participants were significantly more accurate in the Static (vs. Shuffled) condition for the emotions of disgust, happiness, and surprise and less accurate for the emotions of anger and sadness.

**Table 1:** Paired *t*-test results comparing the accuracy in the Dynamic and the Static conditions, the Dynamic and the Shuffled conditions, and the Static and Shuffled conditions.

		Dynamic/static	Dynamic/shuffled	Static/shuffled
Anger	*t*	6.5	3.4	−2.66
	*p*	<0.001^*^	0.0024^*^	0.0129
	*d*	1.24	0.63	−0.5
Disgust	*t*	8.2	13.8	9
	*p*	<0.001^*^	<0.001^*^	<0.001^*^
	*d*	1.55	2.6	1.69
Happiness	*t*	4.3	7.3	4.9
	*p*	<0.001^*^	<0.001^*^	<0.001^*^
	*d*	0.8	1.4	0.91
Sadness	*t*	5	0.4	−5.71
	*p*	<0.001^*^	0.67	<0.001^*^
	*d*	0.95	0.08	−1.08
Surprise	*t*	5.6	8.7	6.8
	*p*	<0.001^*^	<0.001^*^	< 0.001^*^
	*d*	1.06	1.64	1.29

*Paired t-tests were not performed for fear and neutrality since no significant effect of Condition was found in the one-way ANOVAs*.

* *Significant at a p < 0.003*.

*d = Cohen’s d*.

#### Spatial Frequency Tuning

**Figure 6:**
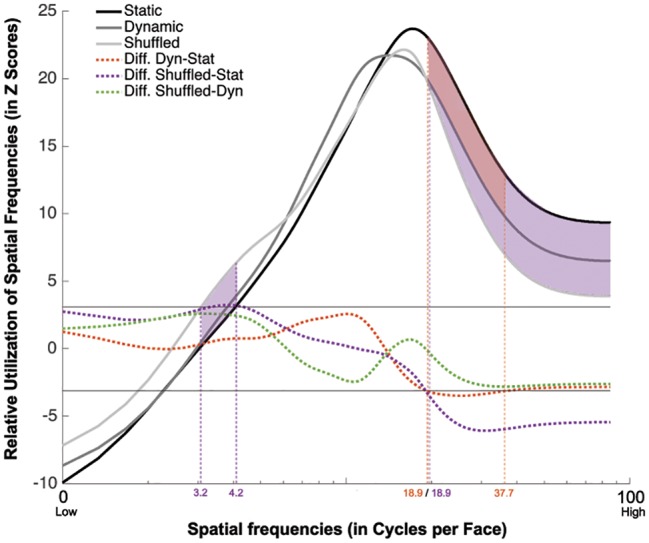
Association between the availability of a given SF and participants accuracy for recognizing static (in black), dynamic (in dark gray), and shuffled (in pale gray) expressions. This association is averaged across all participants and emotions. The dotted red line represents the difference between the Dynamic and Static conditions. The dotted purple line represents the difference between the Shuffled and Static conditions. The dotted green line represents the difference between the Shuffled and Dynamic conditions. The red shaded area indicates the SFs that were significantly less useful in the Dynamic than the Static condition. The purple shaded area indicated the SFs that are significantly more used in the Shuffled than in the Static condition.

The group classification vectors obtained in the Static, Dynamic, and Shuffled conditions were produced using the same procedure as described in Experiment 1. The results are displayed in Figure 6. SF tunings peaking at 17.0, 14.3, and 16.0 cpf with FWHMs of 32.0, 26.7, and 21.0 cpf were found in the Static, Dynamic, and Shuffled conditions, respectively (Z_Crit_ = 3.1, *p* < 0.025).

A significant tuning difference was found between the tunings of the Static and Dynamic conditions: mid-to-high SFs ranging between 18.9 and 37.7 cpf were significantly more useful for static expressions. Significant differences were also found between the Static and Shuffled conditions, whereby low SFs ranging between 3.2 and 4.2 cpf were significantly more useful in the Shuffled condition and SFs higher than 18.9 cpf were significantly more useful in the Static condition. Moreover, no significative differences were found between the SF tuning of Dynamic and Shuffled conditions.

#### Discussion

Although the higher reliance on lower SFs with dynamic than with static expressions observed in Experiment 1 was not replicated, we did find a decreased reliance on higher SFs. This is consistent with the idea of a shift in SF tuning between static and dynamic expressions which will be further discussed in the next section.

A shift toward lower SFs was also observed for shuffled expressions. This suggests that the differences observed in the SF tunings for static and dynamic expressions are not caused by the presence of informative motion. In fact, contrary to what was expected, eliminating or reducing the amount of information contained in the motion by altering the natural sequence of facial changes led to a SF tuning significantly lower than the one observed in the Static condition and similar to the one observed in the Dynamic condition.

## Analysis of Experiments 1 and 2 Combined

**Figure 7:**
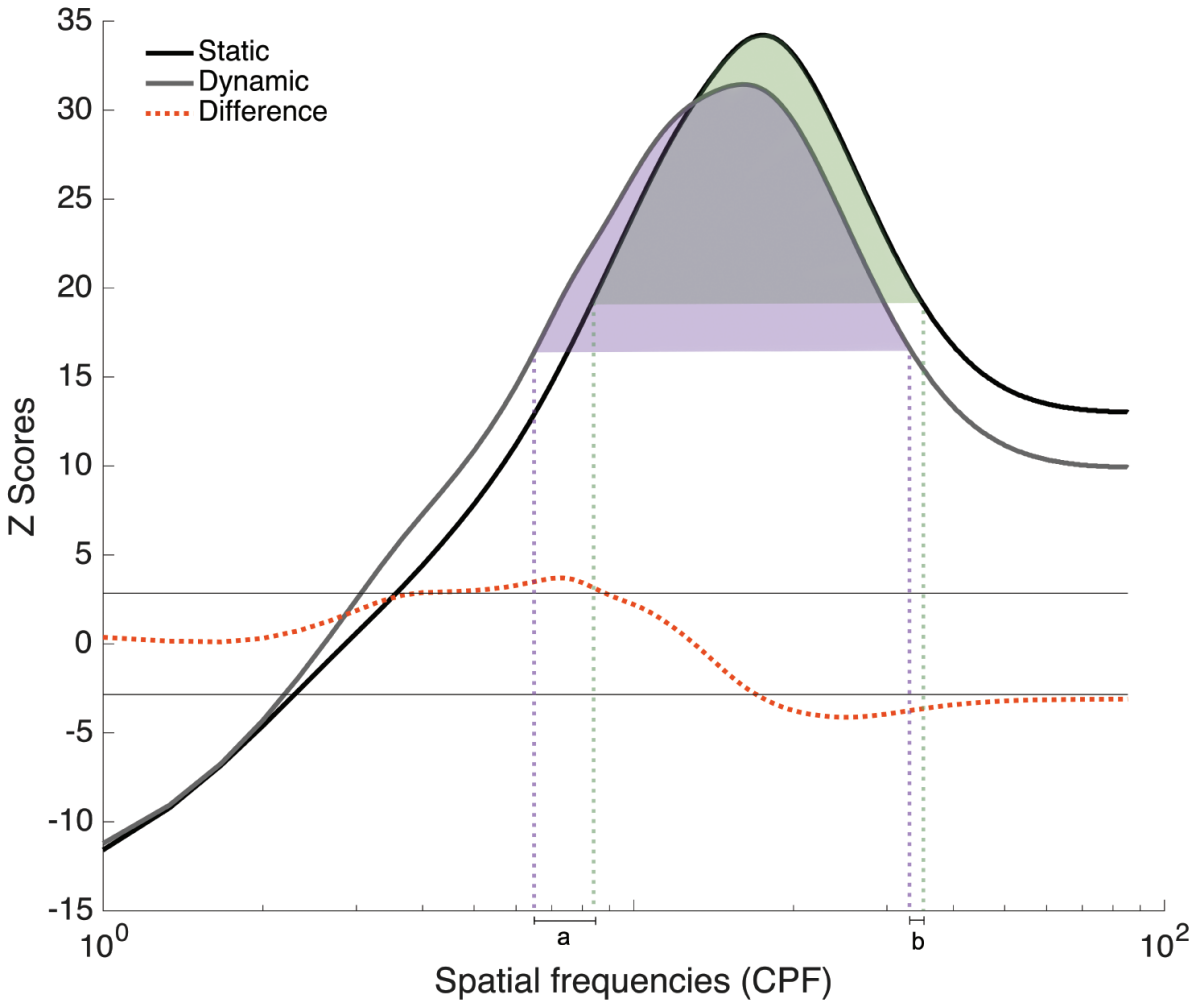
Classification vectors representing the SFs used by the 48 participants tested in Experiments 1 and 2 for static and dynamic facial expressions, averaged across all emotions. The horizontal gray lines represent the statistical thresholds. The dotted red line represents the difference between the Dynamic and Static conditions. The vertical dotted lines corresponded to the beginning and end of each tuning curve at its half maximum (Dynamic in purple and static in green).

Since participants in Experiments 1 and 2 all completed trials with static and dynamic expressions, additional analyses combining all 48 participants were conducted in order to verify the robustness of the SF tuning shift between these conditions. Group classification vectors based on the 48 participants tested in Experiments 1 and 2 were produced for the Static and Dynamic conditions using the same procedure as described in Experiment 1. The results are presented in Figure 7. A SF tuning peak at 17.3 cpf with a FWHM of 31.3 cpf and a SF tuning peak at 16.0 cpf with a FWHM of 28.3 cpf were found in the Static and Dynamic conditions, respectively. Low SFs ranging between 5.6 and 8.3 cpf were significantly more useful in the Dynamic condition and mid-to-high SFs ranging between 17.6 and 85.3 cpf were significantly more useful in the Static condition. Note that the presence of extremely high SFs (i.e., >25 cpf) in the significant clusters is most likely due to the logarithmic SF sampling mentioned in the Materials and Methods; this impacts the resolution of the high SFs, as we have previously demonstrated in a previous study (see supplementary material in Estéphan et al., 2018).

In order to better quantify the tuning shift, we conducted a permutation analysis in which we randomly reassigned the Static and Dynamic conditions during the creation of the group classification vectors. More specifically, on each iteration of the permutation analysis, the Static and Dynamic classification vectors of each participant were randomly assigned to either group classification vector. This procedure was repeated 10,000 times, which allowed us to estimate differences that may have occurred by chance. Two measures were taken: the distance between the tuning peaks for static and dynamic expressions and the translation between the two curves. This last measure was calculated in three steps. First, we indexed the SFs that corresponded to the beginning and end of each tuning curve at its half maximum [Figure 7; purple (Dynamic) and green (Static) dotted lines]. Second, SF values delineating the beginning of the static tuning curve were subtracted from those delineating the beginning of the dynamic curve (see value *a* in Figure 7); and SF values delineating the end of the static tuning curve were subtracted from those delineating the end of the dynamic curve (see value *b* in Figure 7). Finally, these two values, *a* and *b*, were added together. This measure therefore captures differences in the global shape of the tuning curves, as well as their relative position on the SF spectrum, whereas peak displacement reveals differences in SF values to which participants are most sensitive between static and dynamic expressions. For both of these measures, the value corresponding to the 5th percentile across these 10,000 pairs of random classification vectors was used as threshold. In terms of peak displacement, the difference observed between static and dynamic expressions (1.33 cpf) was marginally significant [95% CI (−1.66, 1.66), *p* = 0.0759]. In terms of tuning curve displacement on the SF spectrum, SF tuning for dynamic expressions was significantly translated toward lower SFs (6.33 cpf), relative to static expressions [95% CI (−5.33, 5.66), *p* = 0.02]. Note that this permutation analysis only revealed a significant effect on peaks in Exp. 2 [average of 3 cpf; 95% CI (−3, 3), *p* = 0.05]. There was no significant difference in tuning peaks in Exp. 1 [average of 0.67 cpf; 95% CI (−2, 2), *p* = 0.33]. The tuning translation was neither significant in Exp. 1 [average translation of 4.33 cpf; 95% CI (−6.33, 6.33), *p* = 0.11] nor in Exp. 2 [average translation of 8.33 cpf; 95% CI (−48, 48), *p* = 0.38].

We also conducted an analysis to verify if the shift in SF tuning between dynamic and static expressions is related to the increased accuracy observed with dynamic expressions. We calculated the dynamic advantage in terms of accuracy (i.e., accuracy_Dynamic_ − accuracy_Static_) for each participant separately. We then measured the correlation between the individual dynamic advantage and the shifts in SF tunings (Peak_Dynamic_ − Peak_Static_) and the correlation between the individual dynamic advantage and the magnitude of translation between their tunings. The results indicate that the dynamic advantage was not correlated with any of these two measures: *r*(46) = −0.026, *p* = 0.86 and *r*(46) = −0.015, *p* = 0.92 were obtained for the shift in peaks and the translation of tunings, respectively. Finally, we conducted a preliminary analysis to verify if the SF tuning curves differed between men and women. The results indicated no significant effect of sex on the distance between the tuning peaks for static and dynamic expressions and the translation between the two curves. However, the sample was unbalanced with regards to sex and more research will be necessary to confirm this result.

## General Discussion

The present study investigated the SFs used during static and dynamic facial emotion recognition. In Experiment 1, we found higher reliance on lower SFs for dynamic expressions, whereas we found a decrease in higher SF utilization in Experiment 2. Taken together, these results are consistent with the hypothesized SF tuning shift, i.e., away from higher SFs and toward lower SFs for dynamic emotions.

The SF tuning shift was further assessed in a subsequent analysis that combined data from Experiments 1 and 2, using a permutation procedure. This revealed a marginally significant shift in the peak of the tuning curve for dynamic expressions, as well as a significant translation of the tuning curve itself. However, the fact that this result was nonsignificant when datasets of Experiments 1 and 2 were considered separately suggests that the difference is in fact quite small; hence, this last result should be interpreted with caution until replicated again. In the context of the replication crisis that is often discussed nowadays, new practices have been proposed with regard to how statistical results should be reported and interpreted (Amrhein et al., 2017). When interpreting the result of a replication study, as was done here with Exp. 2, it is recommended to base the comparison on the qualitative profile of the results rather than on the *p*-values or the traditional significance status. That said, the present study described two distinct experiments that generated a similar pattern of results, and this pattern was expected based on the higher sensitivity of the magnocellular pathway to both low SF and motion (Livingstone and Hubel, 1988) and also based on previous eye-tracking results (Buchan et al., 2007; Blais et al., 2017). This, we argue, increases the likelihood that dynamic emotions induce a real shift in SF tuning, however small this shift may be.

Experiment 2 explored if the presence of informative motion in dynamic expressions may be the source of the shift toward lower SFs. In contrast with this hypothesis, the results revealed that altering the information provided by the naturally unfolding motion (i.e., shuffled dynamic emotions) did not eliminate this shift toward lower SFs. In fact, while there was no significant difference in SF tuning for dynamic and shuffled dynamic emotions, there was a significant difference in SF tuning for static and shuffled dynamic stimuli. Specifically, lower SFs were significantly more useful for shuffled dynamic expressions than they were for static expressions, and higher SFs were significantly more useful for static expressions than they were for shuffled dynamic expressions. This suggests that motion increases reliance on low SFs, irrespective of whether the natural unfolding of the expression is preserved or not. This is not however to say that motion was not used to gain an advantage during the recognition of dynamic expressions; in fact, higher accuracy for dynamic expressions may be related to utilization of such information.

As for why a shift toward lower SFs might be induced by motion, one possible – though speculative – explanation pertains to the undoubtedly high importance of motion perception from an evolutionary perspective. As such, the brain has likely developed mechanisms that protect and prioritize processing of motion signals, irrespective of whether this motion conveys information pertinent to a given context or not. Several findings from the literature support this idea. For example, studies have revealed the existence of subcortical pathways, in addition to cortical routes of motion processing, that allow motion perception. Such pathways would explain how visual motion perception can sometimes occur in the cortically blind (Tamietto and Morrone, 2016). Among these subcortical structures is the superior colliculus, a structure known for its role in guiding eye movements (Spering and Carrasco, 2015).

There are also studies indicating that motion processing is suppressed during ocular saccades (Ross et al., 1996), that saccades are suppressed prior to motion processing (Burr et al., 1999), and that rapid motion is better processed in peripheral vision (Tynan and Sekuler, 1982). These mechanisms can inform us as to how prioritizing motion processing should affect eye movements. Indeed, they predict that prioritization of motion processing should lead to saccade suppression (i.e., longer fixations), and a fixation location that allows for parafoveal processing of this information, when motion is detected. As such, fixating a face in its center when viewing dynamic expressions is consistent with prioritizing motion processing. This would also predict central face fixations when viewing shuffled dynamic expressions. In turn, parafoveal processing of diagnostic features may lower the spatial resolution of the visual information extracted.

Finally, it was also shown that processing of low SFs is suppressed during saccades (Burr et al., 1994). Thus, in addition to the fact that features are directly fixated (i.e., processed with highest spatial resolution in the fovea) during the processing of static expressions, the larger number of saccades that is also observed in such conditions may also play a role in lowering visual processing of low SFs and increasing reliance on higher SFs.

A second possible and straightforward explanation for the shift toward lower SFs might be the visual percept itself. Indeed, rapid local changes in time might blur higher SFs as a result of temporal averaging in visual short-term memory (Dubé and Sekuler, 2015). Thus, it may be that high SF information is simply not available to later processing stages in the visual system, leading to a decrease in their use and a commensurate increase in lower SF utilization – i.e., the observed SF tuning shift.

As previously stated, our analysis of accuracies supports the idea that informative motion is beneficial to the recognition of facial expressions. Consistent with this is our observation of a dynamic advantage over a majority of static expressions in both Experiments 1 and 2. Taken together the behavioral results of both experiments add to a growing body of evidence showing that dynamic expressions are often better recognized (Wehrle et al., 2000; Kamachi et al., 2001; Ambadar et al., 2005; Bould and Morris, 2008; Hammal et al., 2009; Cunningham and Wallraven, 2009a; Chiller-Glaus et al., 2011; Recio et al., 2011).

Several studies have found that the dynamic advantage was particularly evident when the physical information contained in the stimuli was either limited in terms of intensity (i.e., expressions not at apex) (Ambadar et al., 2005; Bould and Morris, 2008) or deteriorated in terms of shape, texture, or realism (e.g., photo vs. sketch) (Ehrlich et al., 2000; Wallraven et al., 2008; Cunningham and Wallraven, 2009b). In the present experiment, in addition to physical deterioration associated with the filtering procedure, the presentation time was also constrained (450 ms) in order to respect the natural unfolding of dynamic expressions. This may have favored the emergence of a dynamic advantage. One could even argue that the time restriction is involved in the observation of a dynamic advantage, as most studies that failed to find such an advantage presented their stimuli for more than a 1,000 ms (Gold et al., 2013, 1,059 ms; Fiorentini and Viviani, 2011, ~3,000 ms; Bould and Morris, 2008 ~1,500 ms; Widen and Russell, 2015, ~5,000 ms; Kätsyri and Sams, 2008, until answer). Indeed, such an extended presentation duration might allow a deeper exploration of static stimuli and therefore reducing the relative advantage found for dynamic stimuli.

The results of the second experiment also suggest better recognition of dynamic expressions over shuffled dynamic ones for almost all expressions, with the exception of fear and sadness, for which no significant difference was found. This absence of effect for shuffled expressions of fear and sadness corroborates previous results (Cunningham and Wallraven, 2009a; Richoz et al., 2018). One explanation to this increased accuracy found in shuffled fear and sadness might be attributable to the properties of the stimuli themselves. As reported by various participants, the shuffling of frames might have given the impression that actors performing were either having tremors (in the case of fear) or had their lower lip quivering (in the case of sadness). Again, this general advantage of dynamic expressions over shuffled ones supports the idea that motion containing information facilitates the recognition of dynamic facial expressions. However, our results suggest that the mere presence of motion is nonetheless associated with a shift toward lower SFs and that such shift is not associated with the size of the dynamic advantage.

Despite the obvious limits on ecological validity imposed by an artificial laboratory setting, dynamic expressions such as those used in the present study nonetheless represent a more ecological form of facial expressions compared to the static expressions used in previous research. However, the facial expressions depicted in our stimuli were posed by actors, and posed expressions have been shown to differ from spontaneous expressions with respect to clarity (Matsumoto et al., 2009), achieved intensity (Kayyal and Russell, 2013), and, most importantly, temporal unfolding (Ross et al., 2007; Ross and Pulusu, 2013). As it turns out, these differences between posed and spontaneous static expressions translate as differences in visual strategies in facial feature utilization (Saumure et al., 2018). Future studies should therefore examine the impact of motion on visual strategy variations across posed and spontaneous dynamic expressions.

It should also be mentioned that the samples for both studies were unbalanced with regard to gender. Although there is no clear evidence to suggest that sensitivity to motion differs between females and males (Vanston and Strother, 2017), some anatomical and functional differences have been found in regions of the visual cortex known for motion processing (Amunts et al., 2007; Anderson et al., 2013). Moreover, visual acuity has systematically been shown to be better in males (Burg, 1966; McGuinness, 1976; Ishigaki and Miyao, 1994; Abramov et al., 2012), and males also exhibit higher contrast sensitivity across the entire spatiotemporal domain, especially at higher SFs (Abramov et al., 2012). On the other hand, impact of sex on emotional recognition ability has also been studied, and the evidence favors females over males (e.g., Jenness, 1932; Hall, 1978; Collignon et al., 2010; Derntl et al., 2010; Kret and De Gelder, 2012). It would thus be important for future research to test the impact of sex on SF tuning and on the shift found for dynamic vs. static facial expressions – though our preliminary analysis did not corroborate the presence of sex differences in the SF tuning.

Finally, future studies should be conducted with larger stimuli in order to evaluate the impact of changing the visual eccentricity at which diagnostic information falls on the SF tuning. More specifically, it would be interesting to see if such a change in size would magnify the rather small SF peak shift that was obtained in the present study. It is however important to note that stimulus size alone cannot explain this outcome. In fact, one of our prior work on cross-cultural differences in face identification did reveal a considerably larger SF peak shift (as much as 6.68 cpf) as a function of culture, using face stimuli of similar size (i.e., 256 × 256 pixels) (Tardif et al., 2017).

## Conclusion

Although much neuroanatomical and behavioral evidence suggest that dynamic and static facial expressions of emotion could rely on different perceptual mechanisms, little research has directly compared the visual strategies underlying the recognition of both kinds of expressions. The present research sought to address this shortfall by investigating SF tuning underlying the recognition of both types of expressions. Consistent with our hypothesis, our results suggested a shift toward lower SFs for dynamic expressions in comparison to static ones. This shift is not linked to the presence of natural and informative motion *per se*, but instead appears to be caused by the very presence of motion, notwithstanding the information it conveys. Nevertheless, natural motion does seem to be beneficial to the recognition of facial expressions, since both experiments revealed a dynamic recognition advantage over static or shuffled dynamic expressions. More research will be necessary to better understand the observed shift in SF tuning. One promising avenue is the idea that the mere presence of motion activates mechanisms aimed at prioritizing motion processing and that this in turn affects eye movements and SF processing.

## Ethics Statement

The protocol of this experiment was approved by the Research Ethics Committee of Université du Québec en Outaouais and was conducted in accordance with the Code of Ethics of the World Medical Association (Declaration of Helsinki).

## Author Contributions

CB, DF, MPPD, and CS conceived and designed the experiments. MPPD and CS performed the experiments. MPPD, CB, DF, and JD analyzed the data. MPPD, CB, and DF drafted the manuscript. CS and JD reviewed the manuscript.

### Conflict of Interest Statement

The authors declare that the research was conducted in the absence of any commercial or financial relationships that could be construed as a potential conflict of interest.
